# Sustainable Urban Mobility Plans: implementation process and indicators to evaluate effects on physical activity

**DOI:** 10.1093/eurpub/ckac069

**Published:** 2022-11-29

**Authors:** Romanika Okraszewska, Noah V Peters, Lucia A Reisch, Marion Flechtner-Mors, Carlijn B M Kamphuis, Janine Wendt, Daniel A Scheller, Karolina Konsur, Joanna Żukowska

**Affiliations:** Department of Highway and Transportation Engineering, Faculty of Civil and Environmental Engineering, Gdansk University of Technology, Gdansk, Poland; Department of Politics and International Studies (POLIS), University of Cambridge, Cambridge, UK; El-Erian Institute of Behavioural Economics and Policy, Cambridge Judge Business School, University of Cambridge, Cambridge, UK; El-Erian Institute of Behavioural Economics and Policy, Cambridge Judge Business School, University of Cambridge, Cambridge, UK; Leibniz Chair, Leibniz Institute for Prevention Research and Epidemiology—BIPS, Bremen, Germany; Copenhagen Business School, Department of Management, Society and Communication, Frederiksberg, DK 2000, Denmark; Division of Sports and Rehabilitation Medicine, Department of Internal Medicine, University Hospital Ulm, Ulm, Germany; Department of Interdisciplinary Social Science, Utrecht University, Utrecht, The Netherlands; Division of Sports and Rehabilitation Medicine, Department of Internal Medicine, University Hospital Ulm, Ulm, Germany; Division of Sports and Rehabilitation Medicine, Department of Internal Medicine, University Hospital Ulm, Ulm, Germany; Department of Highway and Transportation Engineering, Faculty of Civil and Environmental Engineering, Gdansk University of Technology, Gdansk, Poland; Department of Highway and Transportation Engineering, Faculty of Civil and Environmental Engineering, Gdansk University of Technology, Gdansk, Poland

## Abstract

**Background:**

Active mobility and public transport increase physical activity (PA) levels. With varying intensity and effectiveness, European cities implement Sustainable Urban Mobility Plans (SUMPs) to spur transport-related PA. Therefore, we aim to examine drivers and barriers to SUMP implementation and assess its influence on PA across European cities.

**Methods:**

We screened policy reports to gain insights into SUMP implementation in one Danish, two German and two Polish cities. Further, we conducted semi-structured interviews with SUMP stakeholders in these cities to explore their experiences with SUMP implementation. Thematic analysis of interview transcripts was applied to identify similarities and differences across cities. To assess the effect of SUMP implementation on PA, we searched for data on indicators of transport-related PA.

**Results:**

All investigated cities are committed to sustainable mobility. Nonetheless, complex institutional structures, the dominant role of motorized traffic as well as complex regional and local policy integration hamper SUMP implementation. Danish, German and Polish cities face different contexts in terms of financing, national guidelines and the prominence of sustainability as a policy objective. Each city adopts unique indicators for monitoring the effects of SUMPs on transport-related PA. The variety of indicators and limited data availability impede a comparative evaluation across cities. Constrained by this restriction, we identified motorization rate, modal split and public transport ridership as suitable indicators.

**Conclusions:**

Local idiosyncrasies need to be accounted for when assessing the implementation of SUMPs. Nonetheless, consistent indicators and data transparency are essential for comparing the effectiveness of SUMPs and their impact on PA.

## Introduction

Through multiple direct and indirect pathways, physical activity (PA) can prevent major non-communicable diseases responsible for premature death and disability.^1^ Transport-related PA can contribute significantly to meeting the World Health Organization’s (WHO) recommendation for daily PA.^2^^,^^3^ Daily PA-levels associated with walking, cycling or use of public transport (PT) are higher than levels gained from driving a car.^4–6^ Reviews suggest PT users may gain an additional 12–15 out of 30 min of daily PA recommended by the WHO.^5^

Sustainable Urban Mobility Plans (SUMPs) are a comprehensive planning tool for promoting active mobility and PT.^7^ To improve quality of life, SUMPs are designed to meet the mobility needs of residents, businesses and other urban stakeholders. Each SUMP caters to a particular city and consists of a mix of policies and measures. Although often not explicitly defined, SUMPs aim at linking transportation and health. By promoting active mobility and PT, SUMPs may contribute to increasing transport-related PA.^8^ Thus, these city-wide policies can be important instruments for boosting PA.

For several years, the European Commission has strongly recommended that cities across Europe implement SUMPs. However, the status of SUMP development and implementation varies widely between countries and cities. Despite existing practitioner-oriented resources,^7^^,^^9^^,^^10^ the scholarly literature on the processes, facilitators and barriers of SUMP implementation is more limited. Evidence on the possible impact of SUMP implementation on PA-levels is largely lacking. Therefore, the present study pursues two goals. First, we aim to illuminate the drivers and barriers European cities face when implementing SUMPs. Second, we seek to evaluate the effect of SUMP implementation on PA-levels.

## Methods

### City selection

We selected five cities in three European countries based on the composition of our research team within the consortium^11^ and language considerations: Copenhagen (Denmark), Gdynia and Wroclaw (Poland) as well as Stuttgart and Ulm (Germany). The countries differ considerably in terms of SUMP implementation. Denmark and Germany are regarded as active SUMP adopters, whereas Poland—previously considered an engaged country—has recently become inactive.^9^^,^^12^ Consequently, the three countries exhibit different motives for implementing SUMPs. Denmark boosts a demonstrated history of sustainable and active mobility, particularly cycling.^13^^,^^14^ In Poland and Germany, SUMPs mark a novel pivot towards sustainable mobility planning.

In Denmark, the national Ministry of Transport helms mobility planning, whilst municipalities are responsible for transport and land-use policies. Denmark’s SUMP strategy caters to the Danish context while also drawing upon European guidance.^15^ Several cities have developed local plans, and Copenhagen and Odense implement second-generation SUMPs.^9^ Copenhagen developed its SUMP in 2012 and presented an update in 2017.^16^^,^^17^

In Poland, the 2010 Public Transport Act established urban mobility planning on a national level and requires cities to pass a plan for sustainable urban mobility.^18^ In absence of a legal obligation to implement SUMPs and limited national guidance, the Ministry of Development Funds and Regional Policy initiated information campaigns and support programmes for cities and has recently launched a pilot programme for regional co-operation targeting cities and functional areas. Nevertheless, our Polish case cities developed their respective SUMPs based on EU guidelines and, in the case of Gdynia, within the CIVITAS DYN@MO (2012–16) framework. The City of Gdynia adopted its SUMP in 2016, and the City of Wroclaw introduced its plan in 2019.^19^^,^^20^

Rather than using the term ‘SUMPs’, German municipalities refer to their respective programmes as ‘transport development plans’ (‘Verkehrsentwicklungsplan’). Nonetheless, most municipalities engaged in SUMP development have fundamentally reorganized previous transport-planning frameworks to embrace sustainable mobility concepts. In 2014, the Stuttgart City Council adopted the ‘Transport Development Concept 2030’, which recommends modifications in all transport sectors.^21^ Building on its traffic development plan originally adopted in 1995, the City of Ulm now deploys its ‘Traffic Development Plan Ulm/Neu-Ulm 2025’.^22^ German federal and state governments support municipalities in implementing sustainable mobility. The Federal Environment Agency provides municipalities with scientific advice and guidance.^23^ Embracing SUMPs also allows German municipalities to tap into new funding schemes.^24^

### Evaluating SUMP implementation: drivers and barriers

As each city’s SUMP is embedded in a unique cultural and institutional context, we first conducted an in-depth analysis of the facilitators and barriers in SUMP development and implementation. Exploiting selected items from previous SUMP evaluations,^9^^,^^10^ we compiled a catalogue of questions for desk research and interviews in each city. Moreover, we added two new items on cities’ general approach to sustainability and how SUMPs consider the needs of vulnerable populations. Lastly, we arranged our questionnaire in a novel way, informed by the evaluation science literature.^25^^,^^26^ This way, our catalogue of questions captures the major steps of the policy implementation process. The questionnaire is available in the Supplementary material.

Having crafted our evaluation framework, each local team addressed the questions by consulting relevant policy reports on SUMP implementation and related city documents. Within city administrations, only one or a small number of staff are responsible for SUMPs. We contacted these individuals and asked them to participate in the study. In addition, we recruited practitioners in urban planning consulting, academia and civil society familiar with local SUMP adoption (purposive and snowball sampling).

Each local team conducted semi-structured interviews independently between late spring and autumn 2020, partially online (due to the Covid-19 pandemic). Because our participants are experts in their respective fields, the semi-structured approach allowed us to elicit qualitative detail of SUMP implementation and mobility policy in general. Apart from the interviews with Copenhagen representatives, whom we interviewed in English, interviews in Germany and Poland were conducted in the respective native languages. We informed participants about the purpose of the study and acquired informed consent.

To conduct interviews, we drew on our uniform catalogue of questions. Having already addressed multiple items during desk research, we focused on a smaller number of questions. Items on concrete drivers and barriers, cities’ idiosyncratic planning traditions as well as SUMP evaluation required deeper insights, and our initial document analysis could not sufficiently elucidate these aspects.

Having completed and audio recorded the interviews, we transcribed the recordings intelligent verbatim and anonymized our participants. To analyse the data, we used thematic analysis. Following Braun and Clarke’s approach, we developed thematic maps encapsulating SUMP implementation in each city of interest (see Supplementary material).^27^ We employed a combination of inductive and deductive coding. Themes could arise naturally from the data (inductive coding), revealing the local contextual factors we are interested in. Simultaneously, we kept track of our questionnaire (deductive coding) to address important implementation domains.

### Pre-post analysis

Our second aim was to apply a pre-post analysis of existing data to evaluate the impact of SUMP implementation on city residents’ PA-levels. For this purpose, we searched for three sets of indicators assessing, either directly or indirectly, the impact of SUMP implementation on PA: (i) indicators (goals) extracted from the five cities’ SUMPs, (ii) ten WHO indicators specifically developed for evaluating PA policies^28^ and (iii) additional indicators retrieved from other SUMP-related resources, e.g. reports on local transport policy.

To evaluate cities’ SUMP impact, we followed two approaches. For indicators adopted from the respective SUMPs, we compared baseline values (pre-SUMP implementation) and follow-up values (post-SUMP implementation) with relevant target values mentioned in the documents. For the WHO and additional indicators, target values were typically not available because the SUMPs did not refer to these measures. We tried to gather relevant information from SUMPs and other resources to compare baseline values with follow-up information.

To evaluate the indicators, we identified overarching topics (e.g. PT infrastructure, road safety) and, depending on the outcome, applied a positive, negative, or neutral trend label to each indicator. Having evaluated each city programme, we compiled a list of common indicators. Aiming to conduct a *comparative* assessment, we were mainly interested in indicators for which information was available across cities.

## Results

We achieved four interviews in Copenhagen, three in Gdynia, two in Wroclaw, two in Stuttgart and one in Ulm (table 1).

**Table 1:** Summary of interview partners

City	Country	Number of interviews and stakeholder categories
Gdynia	Poland	3 (1 PM&G, 1 RC, 1 SCO)
Wroclaw	Poland	2 (1 PM&G, 1 SCO)
Copenhagen	Denmark	4 (1 PM&G, 2 RC, 1 P&P)
Stuttgart	Germany	2 (2 PM&G)
Ulm	Germany	1 (1 PM&G)

*Notes:* PM&G, policymakers and government; RC, research community; P&P, practitioners and professionals; SCO, civil society organizations.

### Institutional complexities hamper SUMP implementation

Institutional complexities remain one of the prime barriers to SUMP implementation. In Copenhagen, Gdynia, Wroclaw, Stuttgart and Ulm, horizontal and vertical co-ordination severely complicates SUMP adoption. Lord Mayors and other steering groups helm transport policy and major investments, particularly in our Danish and German case cities. Below this upper echelon, specialized departments devise and implement policies but are constrained by political quarrels and the higher tier’s budget considerations. The result is an integrated approach: resources, competencies and responsibilities are divided between various tiers of government and departments but need to be harmonized to drive SUMP implementation. Such processes are lengthy, require intensive communication, high headcounts and financial resources.

Our Polish cities represent two diverging organizational approaches: The Wroclaw city administration clusters major competencies around one organizational unit, whereas Gdynia delegates responsibilities to various departments. Both approaches converge to similar political dynamics. Lengthy political negotiations spurred by competing priorities and the complexity of SUMPs constrain project implementation. Even if planning domains are consolidated in one department, the SUMP process requires that various legal, financial and political considerations be harmonized.

This is particularly true for funding. Leaving aside the specifics of each city, finance must be pieced together by combining various funding sources. Often, each project has its own funding needs. Except Stuttgart, the cities we studied do not have comprehensive SUMP budgets. Assembling project funding complicates the implementation process in these cities.

In addition to institutional complexities within cities, co-operation with higher-level entities as well as knowledge sharing determine SUMP implementation. This exchange is crucial for all parts of the SUMP process, from development to implementation. Nonetheless, involving higher-level agencies also obscures SUMP implementation. To varying extents, this observation pertains to all case cities. For instance, metropolitan areas like Greater Copenhagen and Stuttgart liaise with neighbouring municipalities. Ulm sits on the state border between Baden-Württemberg and Bavaria and needs to consider different state laws when devising SUMP initiatives. While Gdynia, Gdansk and Sopot, the core of a multi-communal metropolitan area, have so far proceeded with individual SUMPs, the cities see a burgeoning interest in regional co-operation and develop a metropolitan SUMP.

European guidance is vital for the Polish cities, which have only recently begun to embrace SUMP development. SUMP adoption in Gdynia and Wroclaw hinges almost entirely on EU guidelines and training materials. They also play an important yet less pronounced role in Stuttgart and Ulm. In contrast, Copenhagen mostly draws on municipal resources and extensive experience at the local level. Polish representatives also expressed a keen interest in national guidance complementing European materials and training. Gdynia and Wroclaw as well as Copenhagen policymakers concentrate on municipal efforts because of a (perceived) lack of national support.

Ironically, national legislation often constrains municipal initiatives, e.g. environmental zones in Copenhagen. Consequently, national regulation seems to hamper rather than support local SUMP adoption in Poland and Denmark. This picture is slightly different in Germany. Although traditionally decentralized, the federal government has increased national support for SUMP adoption, a provision utilized by policymakers in Stuttgart and Ulm.

### Sustainability spurs holistic planning but requires institutional reform

Growing awareness at all levels of policymaking aligns with a fundamental shift towards sustainability and urban liveability as leitmotifs of transport policy. Representatives from all sites cited sustainability and urban liveability as important drivers of SUMP implementation, but to varying extents. For instance, creating liveable, environmentally friendly cities has long been a priority in Copenhagen, whereas German and Polish cities are just joining the bandwagon. Many Copenhageners are familiar with sustainability and liveability. Conversely, Polish city administrators had to prop up campaigns to disseminate how cities can become more amiable and environmentally friendly.

This conceptual fundament bears implications for actual transport planning. Copenhagen, Ulm and Stuttgart adopt sustainability as an overarching planning theme guiding SUMP implementation. In Copenhagen, policymakers praise this holistic approach as a new vision for integrated mobility planning. German representatives, however, expressed some frustration with the processual nature of the implementation cycle. Conceiving of all interventions as one coherent whole creates interdependencies and requires rigorous planning, co-ordination and evaluation. Conversely, Polish respondents lamented a lack of holistic vision. SUMP initiatives need to be integrated more clearly with other planning domains. All cities investigated in the present study reassess how they can embrace an all-encompassing mobility theme by creating new and co-ordinating existing departments.

Institutional reform faces its own difficulties. To retain vested interests and privileges, individual stakeholders might be incentivized to maintain the status quo and block extensive reshuffles. Insights from all our case studies in Denmark, Germany and Poland indicate that this reasoning already informs planning practice. Transport decisions are often seen as zero-sum games: extending cycling lanes requires limiting car space; but sustaining the prerogative of the car was referenced as a dominant barrier to SUMP implementation in all case cities.

To break resistance and establish new transport habits, participatory approaches are critical. In Copenhagen, Stuttgart and Ulm, public consultation is a central component of SUMP adoption. Polish cities currently try to devise rigorous public-engagement strategies. While residents were consulted during SUMP development, project implementation has yet to benefit from public input. The Polish example also shows that cities require training and experience to leverage participatory approaches. Involving diverse status groups, especially vulnerable populations, remains a challenge.

### SUMP evaluation varies across cities

#### Copenhagen

Copenhagen’s SUMP sets out 31 implementation goals. Four goals were not included in the original 2012 SUMP but added in the 2017 update. In our pre-post analysis, we assessed all 31 SUMP indicators as well as the 10 WHO indicators by consulting the 2017 SUMP update, Copenhagen’s 2012, 2014, 2016 and 2018 ‘Bicycle Accounts’ as well as the 2013, 2014, 2015 and 2016 ‘Climate Accounts’.^17^^,^^29–37^

Six SUMP indicators refer directly to PA, whilst 18 goals are only indirectly related to PA, and seven indicators have no explicit association with PA outcomes. Furthermore, we were able to assess 7 of the 10 WHO indicators (5 indirect and 2 direct PA indicators). Looking at all indicators together, 10 goals were achieved, that is, met the target value upon evaluation, whereas 8 goals were missed. Twelve indicators could not be evaluated because of missing data. A further eleven indicators require future assessments because the city’s final evaluation is only due in 2020 or 2025, and data are not yet available.

#### Gdynia and Wroclaw

Gdynia’s SUMP defines 24 indicators for monitoring programme implementation. Four of them directly measure PA outcomes, while 18 refer indirectly to PA. Two indicators have no explicit association with PA effects. We acquired information related to 4 out of the 10 WHO indicators. Amongst these, 2 indicators were classified as direct measures of PA. Moreover, we slightly modified the WHO indicator on active school commuting in accordance with the data we could gather. Having screened resources from the city’s statistical office as well as local project and campaign reports, we derived 9 additional indicators, including 5 direct measures of PA effects.

Overall, Gdynia achieved 24 goals (target values were met or positive trend), whereas 3 goals were missed, including 2 SUMP targets (motorization rate, modal share of PT). Because of missing data, we could not evaluate the remaining 14 indicators.

The Wroclaw SUMP comprises 39 indicators, including 2 direct and 1 less relevant measure of PA. We acquired data for 3 WHO indicators, including two directly referring to PA. Based on data from additional sources, we expanded the list by 6 indirect PA indicators. Having compared target values and evaluation outcomes, we found that Wroclaw achieved 26 goals, whereas 20 were missed. Eight targets could not be evaluated due to missing data.

#### Stuttgart and Ulm

To gather indicators for analyzing SUMP effectiveness in Stuttgart and Ulm, we asked relevant city departments to provide information. Moreover, we used Stuttgart’s transport development plan VEK 2030,^21^ the regional transport plan,^38^ resources from Stuttgart’s statistical office and Stuttgart police. Furthermore, the city administration kindly provided additional information.

The Stuttgart SUMP comprises 9 fields of action and associated work packages. The fields of action consist of 63 measures. Implementation indicators were not explicitly specified. To perform a pre-post analysis and comparison with the remaining case cities, we extracted 24 indicators from the SUMP. Specifically, we used Gdynia’s list of indicators as a benchmark without extracting all potential indicators from the Stuttgart VEK. In addition to indicators derived from the Stuttgart document, we obtained information on 4 of the 10 WHO indicators. Due to inconsistent survey approaches, we were limited in comparing these indicators with data from previous years. Any changes can be hardly interpreted.^39^ For Ulm, only the modal split for the period from 2008 to 2017 could be provided.

#### Cross-country findings

Overall, we did not find a comprehensive set of common indicators allowing us to assess the impact of SUMP implementation *across* cities. Based on our heterogeneous findings, we highlight 3 indicators appropriate for comparing the effect of SUMP implementation on PA across cities: motorization rate, modal split and PT use (table 2). The Supplementary material provides a detailed overview of all indicators.

**Table 2:** Selected indicators for evaluating SUMP effectiveness across cities

Indicator	City	Initial value (year)	Target	Follow-up value (year)	Evaluation (trend)
Motorization rate (passenger cars/1000 inhabitants)	Copenhagen	224 (2010)	N.A.	195 (2014)	Positive
	Stuttgart	453 (2010)	N.A.	484 (2020)	Negative
	Gdynia	542 (2015)	550	628 (2018)	Not achieved
	Wroclaw	524 (2011)	Decrease	689 (2018)	Not achieved
Modal share (proportion of transport modes)	Copenhagen	(2010):		(2016):	Not achieved
		Bicycle: 33%	Bicycle: ≥33%	Bicycle: 35%	
		PT: 27%	PT: ≥33%	PT: 22%	
		Car: 40%	Car: ≤33%	Car: 43%	
	Stuttgart	(2010)		(2017)	Not achieved
		Bicycle: 5%	Bicycle: ≥25%	Bicycle: 8%	
		Walking: 26%		Walking: 29%	
		PT: 24%		PT: 23%	
		Car: 45%		Car: 40%	
	Ulm	(2008)	N.A.	(2017)	N.A.
		Bicycle: 11.4%		Bicycle: 12%	
		Walking: 23.3%		Walking: 30%	
		PT: 15.5%		PT: 13%	
		Car: 49.8%		Car: 45%	
	Gdynia	(2015)	N.A.	(2018)	N.A.
		Bicycle: 1.8%		Bicycle: 2.1%	
		PT: 39.8%		PT: 37.1%	
		Car: 57.8%		Car: 49.4%	
		Other: 0.5%		Walking: 11.4%	
	Wroclaw	(2018)	Non-car shares:	N.A.	N.A.
		Bicycle: 6.3%	60% (2020)		
		Walking: 24.2%	65% (2024)		
		PT: 27.6%	70% (2028)		
		Car: 41.4%			
		Other: 0.5%			
		Non-car^a^: 58.6%			
Public transport use	Copenhagen (change in ridership compared to 2011)	N.A.	2% (2015)	2% (2014–15)	Positive
			20% (2025)	N.A. (2025)	
	Stuttgart (rail/bus passengers per day)	(2016)	(2025)	(2018)	Positive
		Rail: 416 000	≥20% increase	Rail: >500 000	
		Bus: 179 000		Bus: 180 000	
	Gdynia (PT trips per capita per year)	240.2 (2018)	Increase	N.A.	N.A.
	Wroclaw (PT passengers per year)	207mn (2018)	Increase	209.6mn (2019)	N.A.^b^
				121.3mn (2020)	

mn = millinons.

a Non-car: Cycling, walking, public transport.

b As of 2020, the coronavirus pandemic has likely affected PT ridership and exacerbates comparisons over time.

## Discussion

In line with previous assessments,^9^^,^^10^ our findings show that co-operation between various local, regional and national agencies remains a prominent barrier to SUMP implementation. A central government unit might alleviate this issue by virtue of more efficient communication and a cohesive organizational culture. Nonetheless, the inherent complexity of cutting-edge mobility policy requires trade-offs between policy objectives. To facilitate such decisions, European SUMP guidance is crucial. Our study inclines us to conclude that European resources are particularly relevant for newly adopting cities, whereas experienced administrations harness local wisdom, tailored to a city’s unique context.

Both European guidelines and local expertise cannot substitute national resources. As evidenced by our findings from Stuttgart and Ulm, national transport guidance and dedicated funding can spur local SUMP implementation. If, however, national legislation constrains cities’ remit to shape transport policy autonomously, local SUMP implementation can falter. To be implemented successfully, SUMPs require a renegotiation of local, regional and national competencies in the realm of transport, land use and energy planning.

While SUMPs are often deployed as stand-alone planning frameworks,^7^ our study shows that support for a broader sustainability transition is decisive. In advanced cities like Copenhagen, urban sustainability can drive SUMP adoption because it is an established motive. In emerging cities, the same theme can ignite SUMP development because it is trendy and novel. At the same time, our results underline existing evidence on the persisting hegemony of the car in European cities.^14^^,^^40^ Car-oriented planning is grounded in culture, nurtured by economic dependencies, and perpetuated by hesitant policymakers. This planning paradigm stirs political stalemate, delays the policy process and undermines integrated mobility concepts.

Consequently, a suitable institutional framework needs to supplement the conceptual underpinnings of SUMPs and any holistic vision. Yet holistic planning strategies are a double-edged sword. On the one hand, complex topics like mobility and sustainability necessitate diverse perspectives, broad political support, and extensive organizational capabilities. On the other hand, such constellations might hamper SUMP implementation because multiple departments and stakeholders jostle for influence and priorities. Institutional streamlining could help create overarching mobility departments, which would exert more far-reaching competencies. Ultimately, such political transitions are driven by the inclinations of the electorate. If policymakers observe a change in residents’ attitudes towards mobility, administrators might embrace extensive reforms.

To which extent policymakers can garner support for sustainable transport remains subject to evaluation. All case cities in Denmark, Germany and Poland engage in some form of SUMP monitoring and evaluation. As eclectic as these approaches are, they are not sufficient in terms of rigour and transparency. Policymakers agree that SUMP interventions need time to materialize but should be evaluated eventually. Identifying unsuccessful interventions is necessary; simply introducing a project does not guarantee success. In general, cities face difficulties in decreasing motorization rates and car traffic, as a share of total modal split. This trend corroborates our qualitative finding that car-focused planning persists.

In any case, evaluation methods need to become more rigorous and transparent. The present study suffers from limited data availability. Cities’ goals and the selection of evaluation indicators are somewhat arbitrary and politically motivated. Available city documents did not allow us to comprehend the measurements’ underlying methodology. We could only use data on motorization rate, modal split and PT use to vaguely compare the influence of SUMP implementation on PA across cities (table 2). Most of the indicators we derived from cities’ SUMPs are indirect measures of PA changes.

Grouping indicators by topic (Supplementary material) shows that cities focus on similar areas of intervention. Because of differences in specific projects indicators for comparing PA effects across cities are limited. For rigorous analyses, consistent indicators, sound methodologies and data transparency are indispensable. The topics we identified demonstrate potential for developing additional uniform indicators to assess indirect changes in PA, e.g. obesity rates, transport education, bicycle and pedestrian network, and PT infrastructure.

Lastly, our small samples restrict the generalizability of our findings. We encourage further studies investigating SUMP implementation in heterogeneous contexts.

## Supplementary data

Supplementary data are available at *EURPUB* online.

## Funding

The PEN project is funded by the Joint Programming Initiative (JPI) ‘A Healthy Diet for a Healthy Life’, a research and innovation initiative of EU member states and associated countries. The funding agencies supporting this work are (in alphabetical order of participating countries): Germany: Federal Ministry of Education and Research (BMBF) (grant numbers: 01EA1818A (for Bremen); 01EA1818D (for Ulm)); Poland: The National Centre for Research and Development (NCBR) grant nr JFA PEN/I/PEN40/02/2019; The Netherlands: The Netherlands Organisation for Health Research and Development (ZonMw) (grant no. 529051020).

*Conflicts of interest*: The authors declare that they have no known competing financial interests or personal relationships that could have appeared to influence the work reported in this paper.

Key points

- Copenhagen (Denmark), Gdynia and Wroclaw (Poland), and Stuttgart and Ulm (Germany) deploy a variety of strategies to implement Sustainable Urban Mobility Plans (SUMPs), which we study and evaluate.
- Sufficient financial resources, horizontal and vertical co-operation between agencies as well as a fundamental emphasis on sustainable transitions are crucial for successful SUMP implementation.
- Motorization rate, modal split and public transport use can be utilized to assess the influence of SUMPs on physical activity.
- Future studies should define clear indicators and collect data to examine the direct impact of SUMPs on physical activity.

## Supplementary Material

ckac069_Supplementary_Data
