# Supplementary material for: Sustainable Urban Mobility Plans: implementation process and indicators to evaluate effects on physical activity
Source: Eur J Public Health. 2022 Nov 29;32(Suppl 4):iv101–6. doi: 10.1093/eurpub/ckac069 (PMC9706112; doi:10.1093/eurpub/ckac069)
Supplement: ckac069_Supplementary_Data [file ckac069_supplementary_data.zip › ckac069_Supplementary_Data/Okraszewska_Impact of SUMPs on PA_SupplMat1_Catalogue_of_questions.docx]

| **EtD-adopted criterion** | **CICI dimension & -domain** | **Code** | **Question providing evidence** | **Answer** | **Source** |
| --- | --- | --- | --- | --- | --- |
| State of adoption | Setting | Q1.1 | What is the city's general approach to sustainability, innovation, and liveability? How are the city's polity and policy integrated regionally, nationally, and internationally? | Free text | New item |
|  | Implementation outcomes | Q1.2 | Which of the categories below regarding SUMP implementation describes best the situation in your city (please tick)? | 1. We have a well-established urban transport planning framework that incorporates SUMPs (or equivalent document), fully supported from the national/regional level with most of the following elements: a SUMP programme, a legal definition, national guidance on SUMPs, assessment scheme, monitoring and evaluation, trainings etc. 2. We have a well-established urban transport planning framework that incorporates SUMPs (or equivalent document) with some support from the national/regional level (see details below) 3. We have an urban transport planning framework that incorporates SUMPs (or equivalent document) without support from the national/regional level – merely as a way of accessing infrastructure funds 4. We are moving towards an approach to sustainable urban mobility planning with very limited or no examples of SUMPs (or equivalent document) 5. Other, please describe | Durlin et al. (2018) |
|  | Socio-cultural context | Q1.3 | What is the average modal split for urban mobility in your city? | Free text | Durlin et al. (2018) |
|  | Geographical context | Q1.4 | How many cities in your country/region have formally adopted a SUMP? | Free text | Durlin et al. (2018) |
|  | Geographical context | Q1.5 | How many cities in your country/region are engaged in the preparation of their first SUMP? | Free text | Durlin et al. (2018) |
|  | Implementation agents | Q1.6 | Which ministry/agency(s) is/are responsible for the urban mobility policy? | Free text | Durlin et al. (2018) |
| Priority of the problem | Political context | Q2.1 | How is urban mobility policy regulated on the local governmental level  (tick as many as apply). | with dedicated programmes,  with dedicated documents,  with specific legislation,  Other, please describe and provide a link: | Durlin et al. (2018) |
|  | Political context | Q2.2 | Are there any major policies supporting preparation and/or implementation of SUMPs in your country/region  (tick as many as apply)? | National / regional transport policy  National / regional cycling policy  Decarbonisation policies/legislation/programmes  Legislation on air quality  Legislation on PT quality / energy efficiency (relative EU Directives only)  Land-use obligations in transport planning  National or regional funding conditional on having a SUMP  National or regional funding conditional on demonstrating that SUMP has been implemented  Others, comments, details: | Durlin et al. (2018) |
|  | Legal context | Q2.3 | Is formal adoption (e.g. in the city council) of a SUMP compulsory in your country/region? Is there any incentive for SUMP adoption? (e.g. legal effects on land use planning, …). Please give details. | Free text | Durlin et al. (2018) |
| Drivers and Barriers | Implementation theory | Q3.1 | Which were the drivers for developing a SUMP in your city? | Free text | Chinellato et al. (2017) |
|  | Implementation theory | Q3.2 | What barriers can you define when it comes to developing and implementing a SUMP? | Free text | Chinellato et al. (2017) |
|  | Geographical context | Q3.3 | How do you think drivers and challenges depend on city structure, geographical location/conditions, national context and maturity in the SUMP process? | Free text | Chinellato et al. (2017) |
| Desirable effects | Implementation outcomes | Q4.1 | What has been achieved by the city programme so far? | Free text | Durlin et al. (2018) |
| Undesirable effects | Implementation outcomes | Q5.1 | What are needs for development or improvement of the existing elements of the city programme? | Free text | Durlin et al. (2018) |
|  | Political context | Q5.2 | Are there any major policies counteracting preparation and/or implementation of SUMPs in your city? | Free text | Durlin et al. (2018) |
|  | Socio-cultural context | Q5.3 | Do you see any gaps in awareness of SUMPs on the higher level of government in your city? (For example, agencies have heard about SUMPs, but do not always understand how they could contribute to better planning / administrators do not know what topics are included and what tools are usually incorporated in SUMPs / there is no body that develops, accesses nor monitors the SUMP preparation and implementation, etc.). | Free text | Durlin et al. (2018) |
| Appreciation and (higher) federal assistance: | Socio-cultural context | Q6.1 | To what extent are agencies in your city familiar with the SUMP or equivalent concept? If several agencies have responsibility for aspects of SUMP, please list each agency and then rank its level of familiarity with SUMP on a “Very familiar” to “Not at all familiar” scale. | Very familiar  Mostly familiar  Some familiar, other not  Mostly not familiar  Not familiar at all  Comments, details | Durlin et al. (2018) |
|  | Implementation process | Q6.2 | Is there any other form of support from the national/regional level to cities which think about, develop or implement a SUMP? Please, provide the link. | Free text | Durlin et al. (2018) |
| Division of responsibility | Implementation strategy | Q7.1 | Are responsibilities divided? If so, which agency has responsibility for what functions and tasks? (For example, who delivers the ideas & vision, who prepares what and who decides on what, are there barriers in their cooperation?). | Free text | Durlin et al. (2018) |
| Resources | Implementation strategy | Q8.1 | Please indicate briefly what resources (if any) are available in your city to prepare a SUMP. | at the local level:  at the regional level:  at the national level:  at the EU level:  other financial resources:  Comments, details: | Durlin et al. (2018) |
|  | Implementation strategy | Q8.2 | Which are main sources of information about the latest development in SUMPs in your city?  Please, provide the link. | Regular SUMP web site?  Newsletter?  Help desk?  Research programme?  Supervisors?  Guidelines?  Other: | Durlin et al. (2018) |
|  | Implementation strategy | Q8.3 | Are regular awareness raising events about benefits of SUMPs and sustainable transport organised on the national/regional level for cities and consultants? If so, who does organise them and how often? | Free text | Durlin et al. (2018) |
|  | Implementation strategy | Q8.4 | Is regular training provided on the national/regional level for cities and consultants involved in SUMP preparation and implementation? | Free text | Durlin et al. (2018) |
| Certainty of evidence of required resources | Implementation strategy | Q9.1 | Is the financial framework for urban mobility secured and clearly defined? | Free text | Durlin et al. (2018) |
| Equity | Socio-economic context | Q10.1 | Does SUMP development consider the needs of vulnerable groups (e.g. ethnic minority populations or people with disabilities) and citizens with low socio-economic status? | Free text | New item |
| Feasibility | Political context | Q11.1 | Is SUMPs development supported from initial steps onwards by coherent guidelines/methodologies adopted, recognised and used at city level? If so, please provide the link. | Free text | Durlin et al. (2018) |
|  | Political context | Q11.2 | Is SUMP development supported by national planning guidelines for specific content of urban mobility policy (e.g. Walking, Cycling, Public transport, Parking etc.)? If so, please list them and provide the link. | Free text | Durlin et al. (2018) |
|  | Implementation process | Q11.3 | Is facilitated knowledge exchange between cities set up in your country/region? If so, who coordinates such a platform? What are other tasks of such a platform? Please, provide the link. | Free text | Durlin et al. (2018) |
| Monitoring & evaluation | Legal context | Q12.1 | Is monitoring and evaluation of the SUMP implementation compulsory in your city? Are indicators for SUMP monitoring and evaluation defined at the city level? Please give details. | Free text | Durlin et al. (2018) |
|  | Implementation outcomes | Q12.2 | Have local monitoring and evaluation schemes been set up to assess the entire SUMP preparation and implementation process? If so, please give details – for example, what information has to be submitted by whom to whom, how often? Who collates all the information? What happens if actors do not supply the information requested? | Free text | Durlin et al. (2018) |

**References**

Chinellato M, Staelens P, Wennberg H, Sundberg R, Böhler S, Brand L, et al. SUMPS-UP. Users’ needs analysis on SUMP take up [Internet]. Brussels, Belgium: European Platform on Sustainable Urban Mobility Plans; 2017. Report No.: D 1.2. Available from: <http://sumps-up.eu/fileadmin/user_upload/Tools_and_Resources/Reports/SUMPs-Up%20-%20Users%27%20needs%20analysis%20on%20SUMP%20take-up.pdf>

Durlin T, Plevnik A, Balant M, Mladenovič L. SUMPS-UP. Status of SUMP in European member states [Internet]. Brussels, Belgium: SUMPs-Up; 2018. Report No.: D 5.1. Available from: <http://sumps-up.eu/fileadmin/user_upload/Tools_and_Resources/Reports/SUMPs-Up_-_SUMP_in_Member_States_report_with_annexes.pdf>

Moberg J, Oxman AD, Rosenbaum S, Schünemann HJ, Guyatt G, Flottorp S, et al. The GRADE Evidence to Decision (EtD) framework for health system and public health decisions. Health Res Policy Syst. 2018;16(1):Article 45.

Pfadenhauer LM, Gerhardus A, Mozygemba K, Lysdahl KB, Booth A, Hofmann B, et al. Making sense of complexity in context and implementation: The Context and Implementation of Complex Interventions (CICI) framework. Implement Sci. 2017;12(1):Article 21.
