# Supplementary figures and images for: Sustainable Urban Mobility Plans: implementation process and indicators to evaluate effects on physical activity
Source: Eur J Public Health. 2022 Nov 29;32(Suppl 4):iv101–6. doi: 10.1093/eurpub/ckac069 (PMC9706112; doi:10.1093/eurpub/ckac069)

# Copenhagen, DK

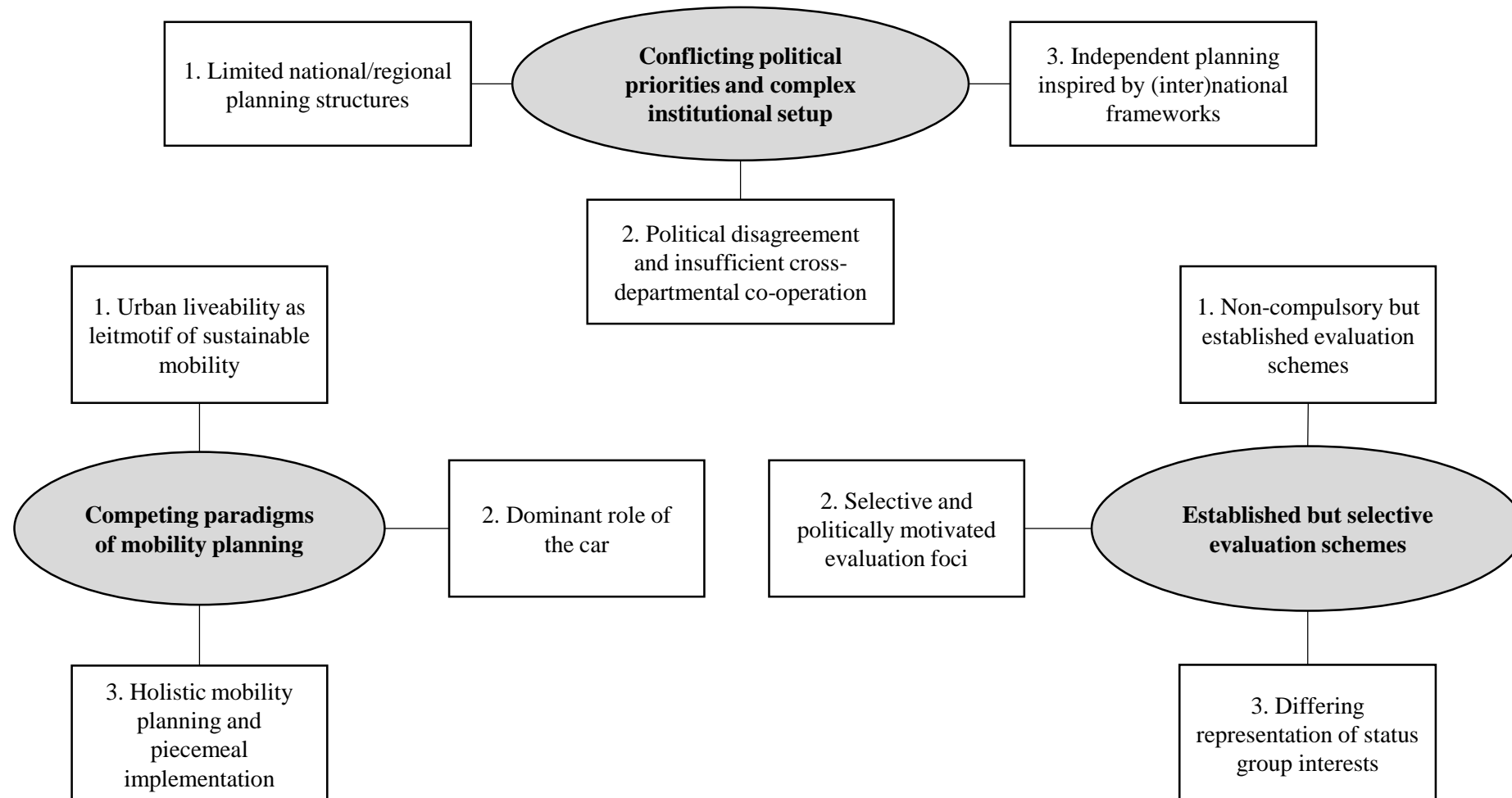

# Stuttgart & Ulm, DE

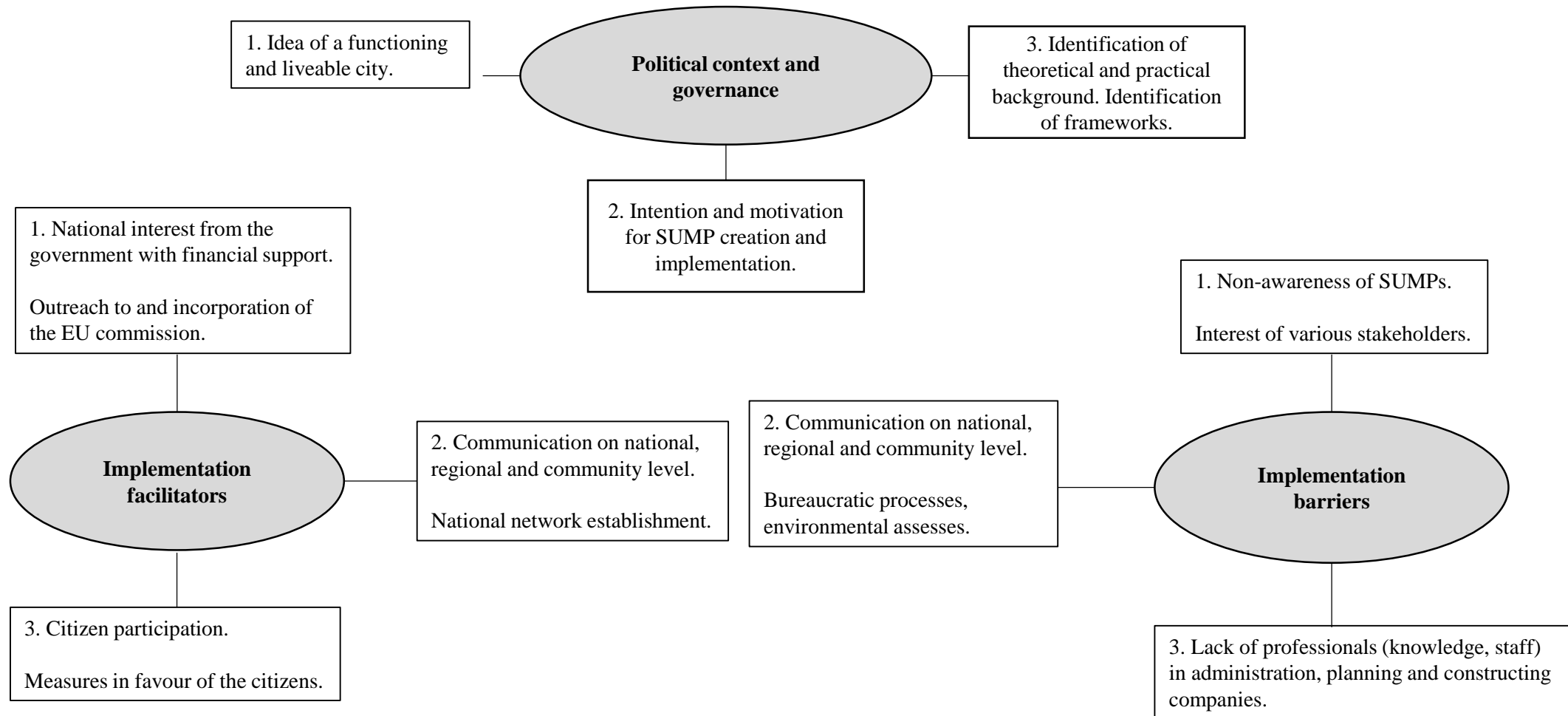

## Gdynia & Wrocław, PL

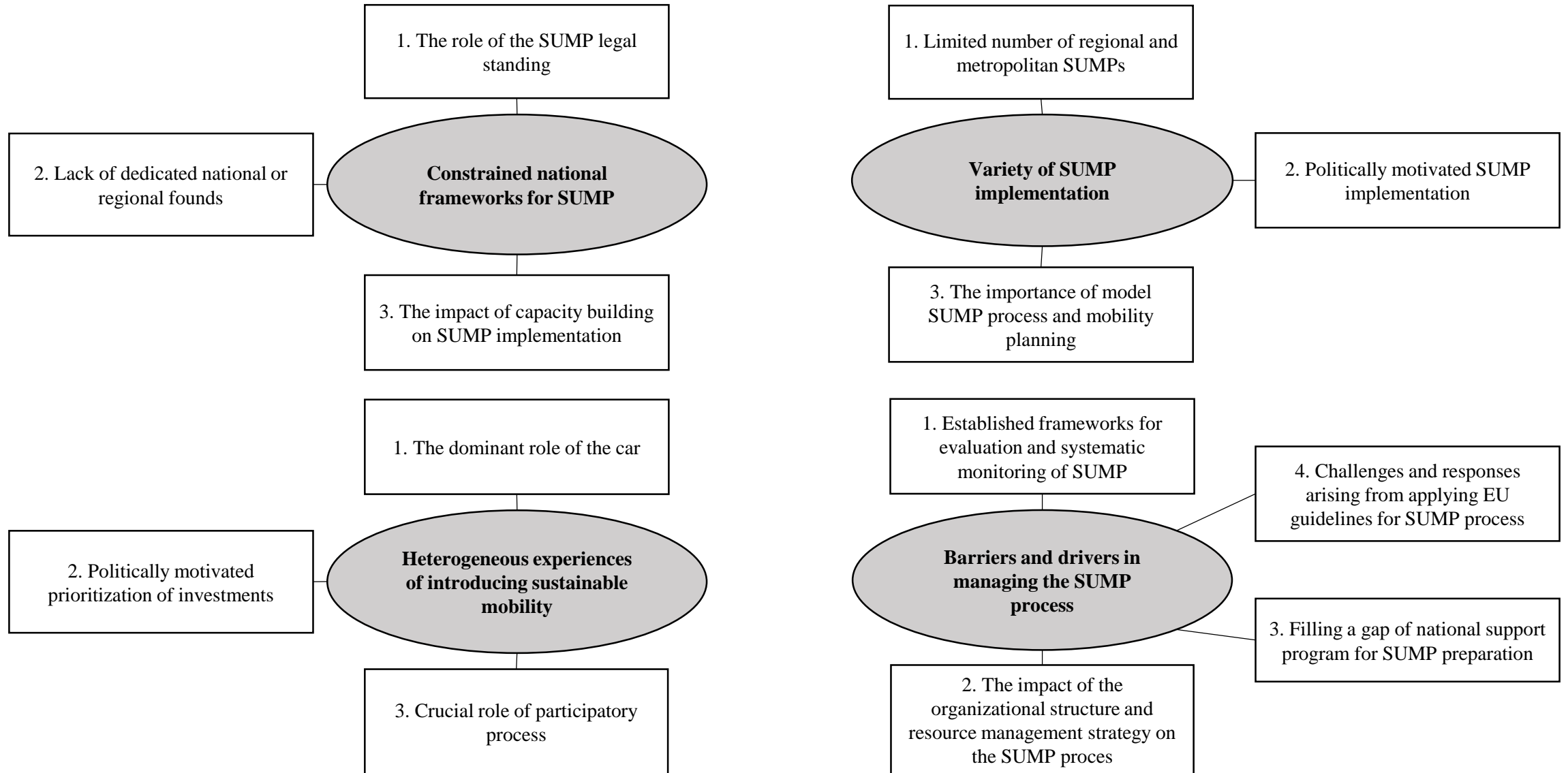

Supplement: ckac069_Supplementary_Data [file ckac069_supplementary_data.zip › ckac069_Supplementary_Data/Okraszewska_Impact of SUMPs on PA_SuppMat3_Thematic_maps.pdf]
